# Point-of-care testing preferences 2020–2022: Trends over the years

**DOI:** 10.1016/j.cvdhj.2024.03.002

**Published:** 2024-03-18

**Authors:** Sakeina Howard-Wilson, Ziyue Wang, Taylor Orwig, Denise Dunlap, Nathaniel Hafer, Bryan Buchholz, Shiv Sutaria, David D. McManus, Craig M. Lilly

**Affiliations:** ∗ Department of Medicine, UMass Chan Medical School, Worcester, Massachusetts; † Manning School of Business, UMass Lowell, Lowell, Massachusetts; ‡ UMass Center for Clinical and Translational Science, UMass Chan Medical School, Worcester, Massachusetts; § Program in Molecular Medicine, UMass Chan Medical School, Worcester, Massachusetts; ‖ Department of Biomedical Engineering, UMass Lowell, Lowell, Massachusetts; ¶ Department of Anesthesiology and Perioperative Medicine, UMass Chan Medical School, Worcester, Massachusetts; ∗∗ Department of Surgery, UMass Chan Medicine School, Worcester, Massachusetts; †† Graduate School of Biomedical Sciences, UMass Chan School of Medicine, Worcester, Massachusetts

**Keywords:** Medical devices, Point-of-care technologies, Laboratory testing, Access to care, Health equity, Insurance, Covid-19, Survey

## Abstract

**Background:**

The use of point-of-care (POC) tests prior to the COVID-19 pandemic was relatively infrequent outside of the health care context. Little is known about how public opinions regarding POC tests have changed during the pandemic.

**Methods:**

We redeployed a validated survey to uncompensated volunteers to assess preferences for point-of-care testing (POCT) benefits and concerns between June and September 2022. We received a total of 292 completed surveys. Linear regression analysis was used to compare differences in survey average response scores (ARSs) from 2020 to 2022.

**Results:**

Respondent ARSs indicated agreement for all 16 POCT benefits in 2022. Of 14 POCT concerns, there were only 2 statements that respondents agreed with most frequently, which were that “Insurance might not cover the costs of the POC test” (ARS 0.9, ± 1.0) and “POC tests might not provide a definitive result” (ARS 0.1, ± 1.0). Additionally, when comparing survey responses from 2020 to 2022, we observed 8 significant trends for POCT harms and benefits.

**Conclusion:**

The public’s opinion on POC tests has become more favorable over time. However, concerns regarding the affordability and reliability of POCT results persist. We suggest that stakeholders address these concerns by developing accurate POC tests that continue to improve care and facilitate access to health care for all.

**Key Points**

• Community members continue to show support for the use of point-of-care tests. All 16 point-of-care test Average Response Scores (ARSs) fell within the "agree" range for POCT benefits.
• When comparing survey responses from 2020 to 2022, significant trends were observed for 5 POCT benefits and 3 POCT harms, likely indicating changing perceptions and concerns among respondents over this period.
• The most commonly agreed upon concern among respondents from 2020-2022 was that insurance might not cover the costs of point of care tests. Respondents in our 2022 survey also expressed agreement with concerns regarding the accuracy of POC tests.

## Introduction

The COVID-19 pandemic foundationally changed American health care and ushered in a new era of telemedicine and remote care.^1^, ^2^, ^3^ The public health response to the pandemic required changes to how individuals sought and received care, especially as it pertained to the use of point-of-care (POC) tests.^4^ POC test use was encouraged by public health professionals and clinicians to facilitate the diagnosis and management not only of SARS-CoV-2 infection, but also of a growing number of other chronic diseases, including cardiovascular, endocrine, respiratory, and metabolic disorders.^5^, ^6^, ^7^, ^8^ Although POC tests have been used for the past several decades, prior use was restricted largely to clinical settings, and it has not been until more recently that POC tests were approved and widely used in the home setting.^4^

Prior research has shown that POC tests are accurate and can be used to improve patient outcomes for several chronic and acute conditions.^9^, ^10^, ^11^, ^12^ POC tests have several advantages over traditional diagnostic tests, including typically having a short time to result, easy administration, and low costs.^11^, ^12^, ^13^ POC tests also have favorably impacted patient outcomes in lower-resource settings.^14^ However, as seen during the COVID-19 pandemic, owing to existing health disparities, not all populations (racial/ethnic, non-English-speaking, uninsured) have been equally able to access, use, and reap the benefits of POC tests.^15^, ^16^, ^17^, ^18^

Although several prior studies suggest that use of accurate, rapid, and affordable POC tests increases consumer demand,^19^^,^^20^ only a few studies have assessed public perceptions of POC tests and almost none have evaluated shifting perceptions of POC tests for a broad array of conditions during the COVID-19 era.^5^, ^19^, ^20^, ^21^, ^22^

Our research aims to fill a knowledge gap by better understanding how individuals perceive use of POC tests for many chronic conditions, including cardiometabolic, pulmonary, and blood disorders. To accomplish this, we administered a validated electronic survey to a large number of community members to assess the extent to which respondents perceive POC tests as useful, to determine which test characteristics they identify as important, and to understand changes to public perception of point-of-care testing (POCT) associated with widespread use during the COVID-19 pandemic by comparing survey responses across all 3 years from 2020 to 2022.

## Methods

### Study population

Survey participants were uncompensated volunteers who were contacted via e-mail for study enrollment. Participants were included in the study if they were >18 years old, were born in the United States, and provided consent to be contacted regarding medical research opportunities. The research reported in this paper adhered to the STROBE guidelines.^23^ Surveys were administered through researchmatch.org and the UMass Chan Conquering Diseases Research Volunteer listserv. Enrollment took place from June 6, 2022, until September 16, 2022. A total of 9296 surveys were distributed. There were 292 surveys received, with a response rate of 3.1%.

### Ethics approval

The University of Massachusetts Chan Medical School Institutional Review Board determined that this study was exempt and waived the requirement for informed consent (docket H00020299).

### Study survey items

As described in our prior research,^19^^,^^20^ we created a validated instrument using qualitative research methods to aid in the development of POCT device design and function through measurement of community member POCT preferences. The validated instrument consisted of 22 questions. Survey questions covered topics on perceived benefits and concerns of POCT and usability. In the development of our 2022 survey, 3 questions were added that addressed the potential benefits of POCT, specifically regarding access to care, availability of testing, and management of care. Responses to most questions were collected and assessed on a Likert scale where participants selected “strongly agree,” “agree,” “neutral,” “disagree,” and “strongly disagree.” Questions addressing ethnicity, age, and race were collected via multiple choice responses.

### Data analysis

Respondent preferences were converted to a 5-point Likert scale (2 = strongly agree, 1 = agree, 0 = neutral, -1 = disagree, -2 = strongly disagree). For every survey item addressing benefits or concerns, mean responses (average response score, ARS) were calculated by multiplying the number of responses received for each corresponding Likert scale option by a numerical Likert scale value. The sum was divided by the total number of item responses. Positive values indicate agreement, and negative values indicate disagreement. A standard general linear model (for continuous variables) was performed to compare the average response scores from years 2020 and 2021 to 2022 using SAS version 9.3. Trend analysis was only conducted for items with data from all 3 years. A *P* value of <.05 was considered significant. To further explore 2022 respondent results, percent agreement was calculated for both concerns and benefits. All responses that indicated “strongly agree” and “agree” were tabulated and categorized as agreement. Responses that were categorized as “strongly disagree” and “disagree” were categorized as disagreement.^24^ Neutral responses were scored as 0 for both average response score tabulations and percent agreement.

## Results

Of 292 surveys received, 285 were completed, resulting in a survey completion rate of 97.6%. As shown in Table 1, slightly more than half of respondents (52.8%) identified as being from the northeastern United States, 21% were from the southeastern United States, 13.4% were from the northwestern United States, 11% were from the southwestern United States, and 1.7% of respondents were from outside of the United States. Of the respondents, over three-quarters (75.2%) identified as female, 22.1% were male, and 2.8% identified as “other” or “would rather not say.” The majority of respondents (86.2%) were white, 14 participants (4.8%) were black, 11 (3.8%) were Asian, fewer than 1% were Native American, and 14 individuals (4.8%) responded “other” or preferred not to respond. The vast majority of survey respondents (89.6%) were aged 31–60 years. There were slightly fewer survey responses in prior study years, including 222 and 267 responses in 2021 and 2020, respectively. The regional and demographic characteristics of survey participants did not differ significantly between 2020 and 2022.

**Table 1:** 2022 Point-of-care benefits and harms survey respondent demographics

Participant demographics	Number (%) of respondents (N = 290)†
**Age (years)**	
Less than 18	0 (0.0%)
18–30	30 (10.3%)
31–60	132 (45.5%)
More than 60	128 (44.2%)
**Sex**	
Female	218 (75.2%)
Male	64 (22.1%)
Other	4 (1.4%)
Would rather not say	4 (1.4%)
**Race**	
Black	14 (4.8%)
White	250 (86.2%)
Asian	11 (3.8%)
Native American	1 (0.3%)
Other	5 (1.7%)
I prefer not to respond	9 (3.1%)
**Ethnicity**	
Hispanic	2 (0.7%)
Latino	3 (1.0%)
Not Hispanic or Latino	285 (98.3)
**Geographic Location**	
Northeastern United States	153 (52.8%)
Northwestern United States	39 (13.4%)
Southeastern United States	61 (21%)
Southwestern United States	32 (11%)
Outside of the continental United States	5 (1.7%)

† Two respondents did not complete demographic survey items.

### Potential benefits of point-of-care technologies

**Figure 1:**
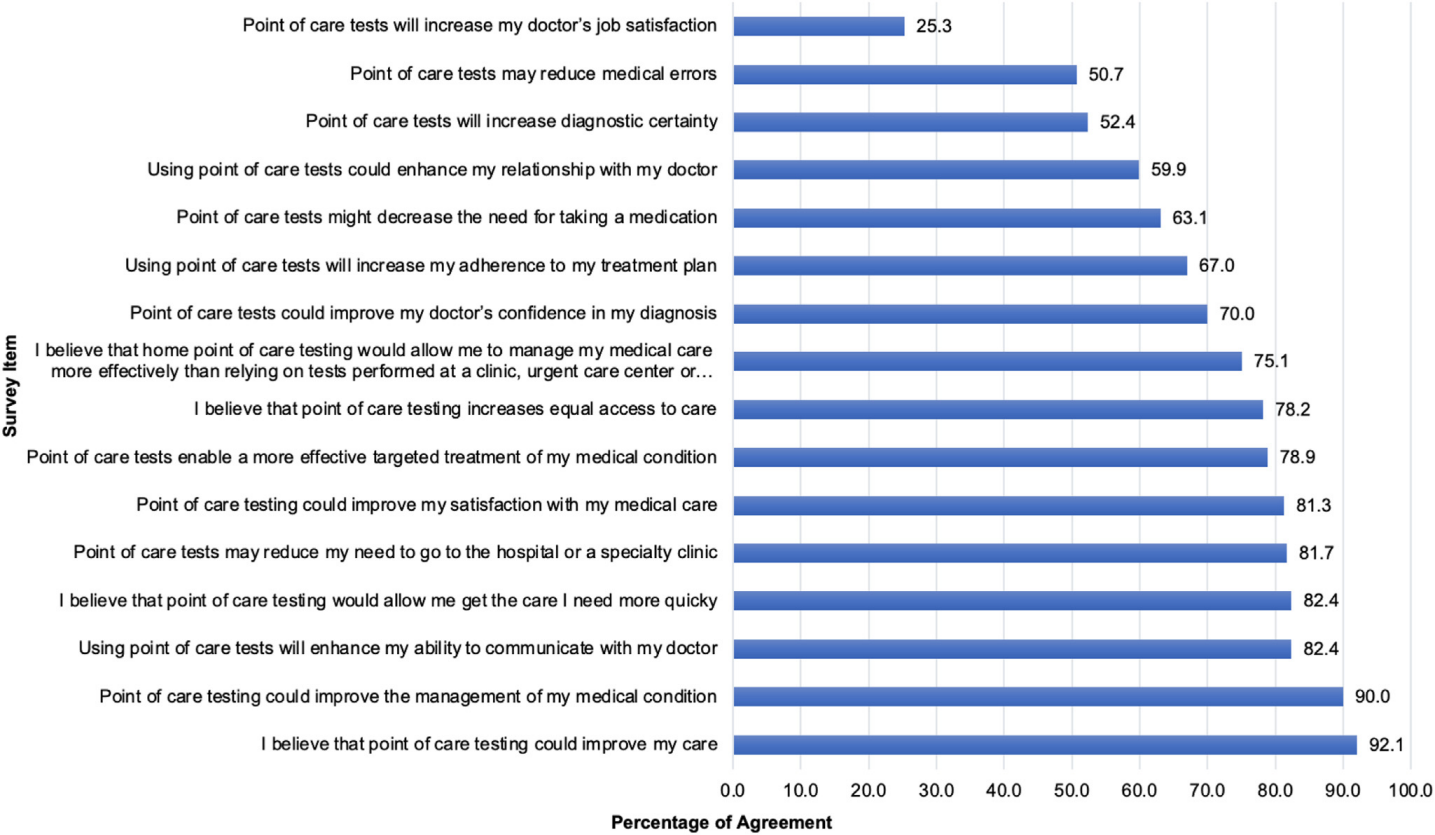
Percent agreement with survey items related to potential benefits of point-of-care tests. Numbers at right indicate percentage of respondents who expressed agreement with statements regarding patient benefits.

Figure 1 shows the responses from participants when surveyed about areas of potential benefit from the use of POC technologies. Table 2 shows the average response scores for 16 survey questions over time from the 3 surveys distributed between 2020 and 2022. In the most recent survey, the statement with the greatest proportion of participants expressing strong agreement was “Point-of-care testing could improve my care,” with 92% agreeing or strongly agreeing (ARS 1.4, ± 0.7). Interestingly, among all 3 years, the POCT benefit that showed the strongest degree of agreement was the belief that “I believe that point-of-care testing could improve my care” (2020 ARS 1.4, 2021 ARS 1.6, 2022 ARS 1.4). Similarly, the proportion of respondents who endorsed the statement “Point-of-care testing could improve the management of my medical condition” was overall unchanged, with 90% of respondents agreeing or strongly agreeing in 2022 (ARS 1.2, ± 0.6), compared to 93% in 2020 and 94% in 2021. However, in contrast to previous years, a smaller proportion of participants in 2022 (52%, vs 68% in 2020 and 66% in 2021) endorsed the belief that “Point-of-care tests will increase diagnostic certainty.” On the other hand, a much higher proportion of 2022 survey respondents (81%, vs 65% in 2020 and 70% in 2021) reported having previously used a POCT. More than 80% of respondents in 2022 showed agreement for the belief that “Using point-of-care testing would enhance my ability to communicate with my doctor” (ARS 1.0, ± 0.7), which was similar to response percentages from prior years ( 80% in 2021 vs 85% in 2020). In our most recent survey, only 1 in 4 survey respondents agreed with the statement “Point-of-care tests will increase my doctor’s job satisfaction” (ARS 0.1, ± 0.8), compared to one-third of respondents in 2020 and 2021 (33% vs 34%).

**Table 2:** Comparison of average response scores regarding potential benefits of point-of-care tests (2020–2022)

Survey item	Average response score			*P* value
	2020	2021	2022	
Point-of-care tests will increase diagnostic certainty	0.7	0.7	0.5	.0198†
Point-of-care tests might decrease the need for taking a medication	0.8	1	0.6	.0020†
Point-of-care tests could improve my doctor’s confidence in my diagnosis	0.8	1	0.7	.0824
Point-of-care testing could improve the management of my medical condition	1.2	1.3	1.2	.4055
Point-of-care testing could improve my satisfaction with my medical care	1	1.1	1	.6490
Point-of-care tests enable a more effective targeted treatment of my medical condition	1	1.1	1	.4035
Using point-of-care tests will enhance my ability to communicate with my doctor	1	1.1	1	.7987
Using point-of-care tests could enhance my relationship with my doctor	0.6	0.7	0.7	.5290
Point-of-care tests may reduce medical errors	0.5	0.6	0.5	.5986
Point-of-care tests may reduce my need to go to the hospital or a specialty clinic	1.2	1.1	1	.0727
Using point-of-care tests will increase my adherence to my treatment plan	0.9	0.9	0.7	.0663
Point-of-care tests will increase my doctor’s job satisfaction	0.3	0.3	0.1	.0268†
I believe that point-of-care testing could improve my care	1.4	1.6	1.4	.5917
I believe that point-of-care testing increases equal access to care	-	-	1.1	-
I believe that point-of-care testing would allow me to get the care I need more quicky	-	-	1.2	-
I believe that home point-of-care testing would allow me to manage my medical care more effectively than relying on tests performed at a clinic, urgent care center, or physician’s office	-	-	1	-

Point**-**of**-**care test average response scores from 2020–2022 for 16 survey benefits are shown. Positive scores suggest agreement, negative scores suggest disagreement. Response categories: 2 = strongly agree, 1 = agree, 0 = neutral, -1 = disagree, -2 = strongly disagree.

† *P* = .05 and suggests a significant linear trend.

### Patient responses to survey items addressing access to care, 2022

Participants were provided with additional survey items in the 2020 questionnaire that addressed POCT benefits regarding access to care, availability of testing, and management of care. More than 82% of respondents agreed with the belief that “Point-of-care testing will allow me to get the care I need more quickly” (ARS 1.2, ± 0.8). Slightly more than three-quarters of individuals (75.8%) agreed with the statement that “Point-of-care testing increases access to care” (ARS 1.1, ± 0.9). Similarly, 75% of respondents agreed with the statement that “Home point-of-care testing would allow me to manage my medical care more effectively than relying on tests performed at a clinic, urgent care center, or physician’s office” (ARS 1, ± 1.0).

### Respondent concerns about point-of-care technologies

**Figure 2:**
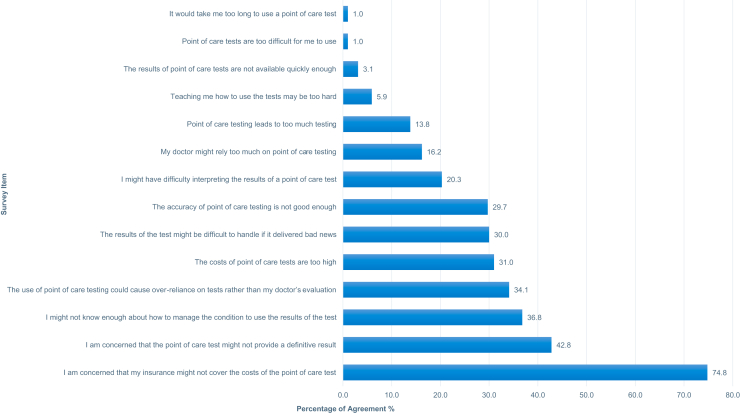
Percent agreement with survey items related to potential benefits of point-of-care tests. The percentage of respondents who expressed agreement to statements regarding patient concerns are shown to the right of the graph.

Figure 2 provides a summary of responses from participants when asked about areas of potential concern about the use of POC technologies. Table 3 shows the average response scores for 14 survey questions over time from the 3 surveys distributed between 2020 and 2022. Most respondents (74.8%) expressed concern that their insurance would not cover the costs of their POC tests (ARS 0.9, ± 1.0). Additionally, 2 in 5 participants expressed concerns that “Point-of-care test[s] might not provide a definitive result” (ARS 0.1, ± 1.0). In our recent survey, these were the only 2 concerns that resulted in a positive ARS overall. The most frequent concern cited by respondents among all 3 years was the belief that “I am concerned that my insurance might not cover the costs of the point-of-care test” (2020 ARS 0.9, 2021 ARS 0.8, 2022 ARS 0.9). Fewer than half of the 2022 respondents agreed with the statements “I might not know enough about how to manage the condition to use the results of the test”( 37%, ARS -0.1, ± 1.2) and “The use of point-of-care testing could cause over-reliance on tests rather than my doctor’s evaluation” (34%, ARS -0.1, ± 1.1). Only a third of participants agreed with the statement “The costs of point-of-care tests are too high” (31%, ARS 0, ± 0.98). The POCT concerns with the smallest proportion of survey participants expressing agreement were “It would take me too long to use a point-of-care test”(1%, ARS -1.4, ± 0.7) and “Point-of-care tests are too difficult for me to use”(1%, ARS -1.4, ± 0.7).

**Table 3:** Comparison of average response scores regarding potential harms of point-of-care tests (2020–2022)

Survey item	Average response score			*P* value
	2020	2021	2022	
Point-of-care testing leads to too much testing	-0.5	-0.7	-0.5	.5181
The accuracy of point-of-care testing is not good enough	0	-0.3	-0.1	.6870
My doctor might rely too much on point-of-care testing	-0.5	-0.6	-0.6	.2882
The costs of point-of-care tests are too high	0.1	0	0	.4753
Teaching me how to use the tests may be too hard	-1.1	-1.1	-1.1	.3601
The use of point-of-care testing could cause over-reliance on tests rather than my doctor’s evaluation	0	-0.5	-0.1	.3130
Point-of-care tests are too difficult for me to use	-1.2	-1.2	-1.4	.0114†
It would take me too long to use a point-of-care test	-1.2	-1.2	-1.4	.0020†
The results of point-of-care tests are not available quickly enough	-0.8	-1	-1.1	.0001†
I might have difficulty interpreting the results of a point-of-care test	-0.3	-0.6	-0.6	.0002†
I am concerned that the point-of-care test might not provide a definitive result	0.2	-0.2	0.1	.4923
I might not know enough about how to manage the condition to use the results of the test	-0.1	-0.4	-0.1	.6502
The results of the test might be difficult to handle if it delivered bad news	-0.1	-0.3	-0.4	.0175†
I am concerned that my insurance might not cover the costs of the point-of-care test	0.9	0.8	0.9	.9116

POC test average response scores from 2020 to 2022 for 14 survey concerns are shown. Positive scores suggest agreement, negative scores suggest disagreement. Response categories: 2 = strongly agree, 1 = agree, 0 = neutral, -1 = disagree, -2 = strongly disagree.

† *P* = .05 and suggests a significant linear trend.

### Trends in survey responses, 2020–2022

Using linear regression to compare survey average response scores for both benefits and concerns, we observed 8 statistically significant changes in average survey responses over the last 3 years (Supplemental Table 1). Between 2020 and 2022, respondents were more likely to disagree or strongly disagree with the following stated concerns, as shown in Supplemental Figure 1: “Point-of-care tests are too difficult for me to use” (*P* = .01), “It would take me too long to use a point-of-care test” (*P* = .002), “The results of point-of-care tests are not available quickly enough” (*P* < .0001), “I might have difficulty interpreting the results of a point-of-care test” (*P* < .002), or “Results might be difficult to handle if it delivered bad news” (*P* = .02). Additional linear regression analyses including data from the potential benefits section of the survey show that respondents had lower rates of agreement in 2022 than in prior survey years for the statements that “Point-of-care tests might decrease the need for taking a medication” (*P* = .002), that “Point-of-care tests will increase my doctor’s job satisfaction” (*P* = .03), or that the use of “Point-of-care tests will increase diagnostic certainty” (*P* = .02).

## Discussion

We observed increasing experience and, overall, increasingly positive views of POCT among survey respondents between 2020 and 2022. Most of the participants who were surveyed in 2022 agreed that POC tests had multiple benefits and expressed few concerns, similar to findings from prior years. Notably, the greatest concern was insurance coverage of the cost of testing. Overall, however, our results suggest that patients favor increasing use of POC tests to diagnose and manage their health.^24^

Prior survey research has suggested that barriers to point-of-care testing include insecurity on the part of patients and clinicians about proper home test use and result interpretation.^21^ However, our results suggest that patients are increasingly familiar with POC tests and express increasing comfort and confidence with their use in the home and clinic setting.

Our results reveal a notable shift in public opinions regarding POCT usage and interpretation ARSs from 2020 to 2022. Respondents were increasingly inclined to disagree with statements concerning various characteristics of POC tests, including their ease of use, convenience, interpretation, and the handling of results. These changes in perception can possibly be attributed to the impact of the COVID-19 pandemic. Although community members have become more familiar with POC tests owing to their widespread use during the pandemic, they have also had more exposure to POC tests. The health care landscape has evolved in response to the pandemic, aiming to better meet patient needs. Instead of POC tests being exclusively offered by physicians in hospitals, they are now accessible through a wide variety of means, including pharmacies, clinics, and even drive-thru health care services provided by various institutions.^25^ In addition to the factors analyzed in our survey, it is worth noting that the rise in chronic diseases in the aging population has encouraged POCT use and exposure. Although not assessed in our survey, this increase in the prevalence of chronic diseases could significantly impact how individuals perceive and use POCT.

One of the major and consistent concerns expressed by survey respondents spanning all 3 years was whether their health insurance would cover the costs of POCT. Although federal mandates ensured that at least some POC tests for SARS-CoV-2 were covered by health insurance plans, most POC tests were not covered for home use.^16^ Our findings suggest that broader POC test adoption is hindered by the fact that insurance plans infrequently pay for the cost of home POC tests for common conditions.^26^ High out-of-pocket costs for POC tests disproportionately impact persons of color or individuals of lower socioeconomic status, and our findings suggest that health care disparities may be exacerbated unless the cost of POCT is fully covered by insurers.^27^^,^^28^ Three-quarters of individuals in our study perceived that POC tests can improve equitable access to care, suggesting that patients desire tests that are deliverable across a wide range of sociodemographic and ethnic/racial categories.

Our results also suggest that patients desire greater accuracy from their POC tests, likely related to their experiences with home antigen tests for SARS-CoV-2.^29^ Between 2020 and 2022, our results have shown that respondents were increasingly likely to disagree with the statement “Point-of-care tests will increase diagnostic certainty.” Additionally, participants strongly agreed with the statement that “Point-of-care testing might not provide a definitive result” in 2020 and 2022, compared to 2021. Our results are consistent with prior studies showing that both patients and health care providers prioritize accuracy when considering use of a POCT for screening or diagnostic purposes.^30^, ^31^, ^32^, ^33^, ^34^, ^35^

### Strengths and limitations

Our study consists of several strengths and limitations. One of the strengths of our study includes the use of a validated instrument administered over 3 successive years spanning the COVID-19 pandemic to assess patient impressions of POC tests. However, our overall survey response rate of 3.1% was low and the overall number of survey respondents was modest. However, this rate is consistent with other studies that used large listservs to deliver electronic surveys.^19^^,^^20^^,^^24^ Most survey participants were female and mostly white women, making our results potentially less generalizable to other racial and ethnic groups. In addition, we included a cohort of patients that had expressed interest in participating in medical research in our e-blast, and thus this group may have greater expertise and different views of POC tests than the general public. Validation of our findings in a large, racially and geographically diverse cohort is needed.

## Conclusion

In conclusion, in our moderately sized but geographically diverse cohort of patient respondents, we observed that the majority of those surveyed reported positive impressions of POC tests for diagnosing and managing their health conditions. We also observed increasing familiarity and comfort with the use and interpretation of POCT results. Although participants support POCT use in the home to manage and improve their care, barriers such as cost and accuracy remain regarding the integration of POC tests into the community. Nevertheless, our findings suggest increasing enthusiasm among patients for using POC tests to better manage their health and health care.
