# Supplementary material for: Point-of-care testing preferences 2020–2022: Trends over the years
Source: Cardiovasc Digit Health J. 2024 Mar 18;5(3):149–55. doi: 10.1016/j.cvdhj.2024.03.002 (PMC11232423; doi:10.1016/j.cvdhj.2024.03.002)
Supplement: Supplemental Figure 1 [file mmc1.docx]

**Supplementary Figure 3**. **Comparison of Average Response Scores from 2020-2022 for Survey Concerns**

**
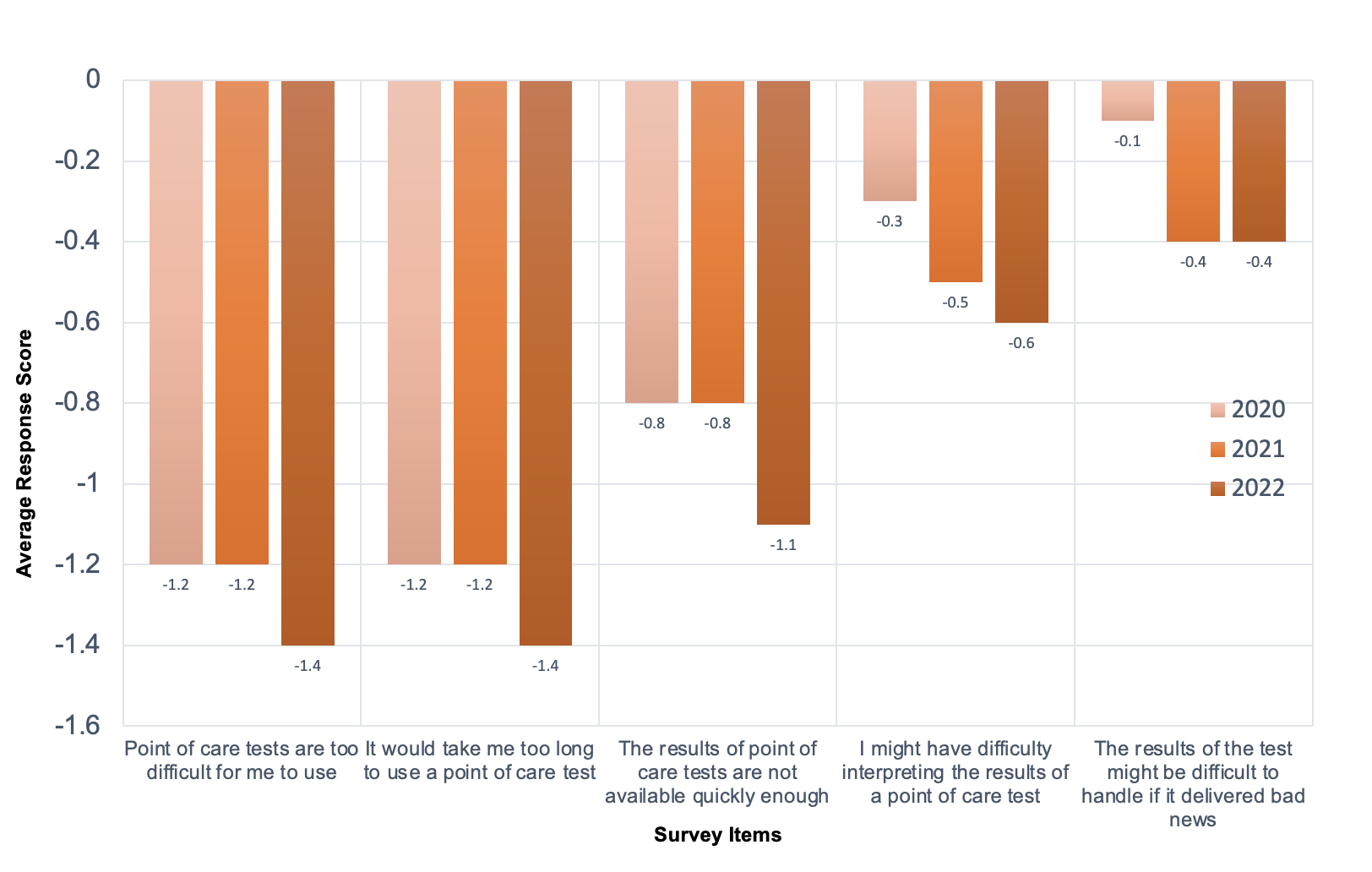
Legend**. Comparisons of Average Response Scores from 2020- 2022 are highlighted above. Average response scores range from 0 (Neutral) to -2 (Strongly Disagree). Responses for 2022 have been mostly negative suggesting greater disagreement compared to years 2020-2021 for concerns above.
