# Supplementary material for: Point-of-care testing preferences 2020–2022: Trends over the years
Source: Cardiovasc Digit Health J. 2024 Mar 18;5(3):149–55. doi: 10.1016/j.cvdhj.2024.03.002 (PMC11232423; doi:10.1016/j.cvdhj.2024.03.002)
Supplement: Supplemental Table 1 [file mmc2.docx]

**Supplementary Table 4: Comparison of Average Response Scores for Concerns and Benefits (2020-2022)**

| Survey Item | Average Response Score 2020 | Average Response Score 2021 | Average Response Score 2022 | $\boldsymbol{\beta}$ | P-value |
| --- | --- | --- | --- | --- | --- |
| Point of care tests are too difficult for me to use | -1.2 | -1.2 | -1.4 | -0.0772 | 0.0114 |
| It would take me too long to use a point of care test | 1.2 | -1.2 | -1.4 | -0.0928 | 0.0020 |
| The results of point of care tests are not available quickly enough | -0.8 | -0.8 | -1.1 | -0.1423 | 0.0001 |
| I might have difficulty interpreting the results of a point of care test | -0.3 | -0.5 | -0.6 | -0.1714 | 0.0002 |
| The results of the test might be difficult to handle if it delivered bad news | -0.1 | -0.4 | -0.4 | -0.1198 | 0.0175 |
| Point of care tests will increase diagnostic certainty | 0.7 | 0.7 | 0.5 | -0.0755 | 0.0198 |
| Point of care tests might decrease the need for taking a medication | 0.8 | 1 | 0.6 | -0.1106 | 0.0020 |
| Point of care tests will increase my doctor’s job satisfaction | 0.3 | 0.2 | 0.1 | -0.0746 | 0.0268 |

**Legend:** A standard general linear model (for continuous variables) was calculated using ARSs from 2020-2022. A negative $\boldsymbol{\beta}$ indicates a trend toward disagreement for the survey questions above.
